# Draft Genome Sequence of the Lactococcus lactis 11/19-B1 Strain, Isolated from Kiwifruit

**DOI:** 10.1128/MRA.00793-20

**Published:** 2020-09-03

**Authors:** Ken Ishioka, Kyoko Nishiyama, Tatsuo Suzutani

**Affiliations:** a Department of Microbiology, School of Medicine, Fukushima Medical University, Fukushima, Japan; University of Maryland School of Medicine

## Abstract

We report here the draft genome sequence of Lactococcus lactis strain 11/19-B1, isolated from kiwifruit. The 11/19-B1 strain possesses one chromosome and five plasmids and has a predicted 2,429 protein-coding sequences. DFAST annotation and a BLASTp homology search estimated that 11/19-B1 possesses three bacteriocin immunity proteins and four bacteriocin proteins.

## ANNOUNCEMENT

Lactic acid bacteria (LAB) are encountered in a wide range of environments, such as animals and plant materials. Lactococcus lactis is one LAB commonly used as a probiotic in the production of many fermented foods, such as yogurts and cheese.

It is reported that the L. lactis 11/19-B1 strain, isolated from the surface of kiwifruit, markedly stimulates innate immunity in silkworms and shows some differences in sugar utilization and enzymatic characteristics in comparison with other L. lactis strains (1). Furthermore, we reported that the intake of yogurt containing 11/19-B1 significantly decreased the level of low-density lipoproteins (LDLs) in high-LDL volunteers in comparison to that of the control yogurt (2). Moreover, 11/19-B1 improved atopic dermatitis (AD) in humans and AD-related dorsal skin and ear lesions in a 1-fluoro-2,4-dinitrobenzene-induced AD mouse model (3). We report here the draft genome sequence of 11/19-B1 used as a starter for commercially available yogurt.

The genomic DNA of laboratory-stocked 11/19-B1 strain cultured at 35°C on sheep blood agar plates was isolated via phenol-chloroform extraction. Genomic DNA was digested with NEBNext double-stranded DNA (dsDNA) Fragmentase (New England BioLabs [NEB]). The digested genomic DNA was then used for construction of an Illumina MiSeq library produced using an Illumina TruSeq Nano DNA low-throughput (LT) kit using the standard protocol. The sequences of the Illumina MiSeq library were determined using MiSeq reagent kits v2 (500 cycles) and v3 (600 cycles). Base calling and quality checking were carried out with MiSeq control software v2.6.2.1 using the default settings, with the resultant number of total reads being 3,406,549. For long-read genomic DNA, the genomic DNA was fragmented with g-TUBE (Covaris), and then short DNA fragments were removed with AMPure XP beads (Beckman Coulter). The fragmented genomic DNAs over 15,000 base pairs long were selected with BluePippin (Sage Science). For construction of the library, the size-selected DNA fragments were treated with NEBNext FFPE DNA repair mix, the NEBNext end repair/dA-tailing module, NEB blunt/TA ligase master mix, and the SQK-LSK108 ligation sequencing kit 1D (Oxford Nanopore Technology [ONT]). The constructed library sequence was determined using a GridION X5 Nanopore sequencer with an EXP-LLB001 library loading bead kit (ONT). The library was loaded onto a FLO-MIN107 (R9.5) flow cell (ONT) and sequenced with MinKNOW v1.7.14 (ONT). Base calling was carried out with Albacore v1.2.4 (https://github.com/Albacore/albacore) using the default settings. The sequence data obtained from the Nanopore sequencer are as follows: total number of reads, 971,849; total number of bases, 9,134,301,538; average read length, 9,398.9 bases; average quality score, 8; *N*_50_ read length, 14,505 bp. The analyzed Nanopore long-read data were used for genome assembly with Unicycler v0.4.6 (4), and the assembled sequence was analyzed with Circlator v1.5.2 using the default settings (5). For trimming the adaptor sequence and removing the low-quality sequence data, the FASTQ sequence data derived from MiSeq sequencing were analyzed with Cutadapt v1.11 (6). The parameters of the Cutadapt software were as follows: overlap, 10 bases; minimum length, 51 bases; quality cutoff value, 30. The Cutadapt-analyzed sequence was then mapped to the contig sequences generated by Circlator using the Burrows-Wheeler Aligner (BWA) v0.7.12 with the ALN (BWA-backtrack) algorithm (7), and the contig sequences were corrected with Pilon v1.22 (8). BWA and Pilon were used with default settings.

The 11/19-B1 strain possesses one circular chromosomal DNA (2,396,965 bases) and 5 circular plasmid DNAs, with the GC content of the total sequence being 35.0%. The DFAST annotation (9, 10) revealed the genomic DNA code consisting of the predicted 2,429 protein-coding sequences containing 707 hypothetical proteins, 19 rRNAs, 65 tRNAs, 1 transfer-messenger RNA (tmRNA), and 2 CRISPRs. These predicted proteins contained three bacteriocin immunity proteins (locus tags LL1119B1_14680, LL1119B1_16170, and LL1119B1_19850). Furthermore, a BLASTp homology search estimated that three lactococcin family bacteriocins (locus tags LL1119B1_02310, LL1119B1_02350, and LL1119B1_02410) and one bacteriocin protein (locus tag LL1119B1_06210) were contained within these 707 hypothetical proteins.

### Data availability.

The sequence data and annotation information of L. lactis strain 11/19-B1 were deposited in the DDBJ/ENA/GenBank database. The accession numbers of the Illumina sequence data are DRX222592 to DRX222595, and that of the Nanopore sequence data is DRX222591. The accession numbers of the genomic sequences and DFAST-analyzed annotations are BLYG01000001.1 to BLYG01000006.1.
